# MicroRNA-195-5p Attenuates Intracerebral-Hemorrhage-Induced Brain Damage by Inhibiting MMP-9/MMP-2 Expression

**DOI:** 10.3390/biomedicines12061373

**Published:** 2024-06-20

**Authors:** Yi-Cheng Tsai, Chih-Hui Chang, Yoon Bin Chong, Chieh-Hsin Wu, Hung-Pei Tsai, Tian-Lu Cheng, Chih-Lung Lin

**Affiliations:** 1 Graduate Institute of Medicine, College of Medicine, Kaohsiung Medical University, Kaohsiung 80708, Taiwan; iiidns11@hotmail.com (Y.-C.T.); chchang20@gmail.com (C.-H.C.); bin99068@hotmail.com (Y.B.C.); 2 Division of Neurosurgery, Department of Surgery, Kaohsiung Medical University Hospital, Kaohsiung 80708, Taiwan; wujoeys@gmail.com (C.-H.W.); carbugino@gmail.com (H.-P.T.); 3 Department of Surgery, School of Medicine, College of Medicine, Kaohsiung Medical University, Kaohsiung 80708, Taiwan; 4 Regenerative Medicine and Cell Therapy Research Center, Kaohsiung Medical University, Kaohsiung 80708, Taiwan; 5 Department of Biochemistry, School of Post Baccalaureate Medicine, College of Medicine, Kaohsiung Medical University, Kaohsiung 80708, Taiwan; tlcheng@kmu.edu.tw; 6 Drug Development and Value Creation Research Center, Kaohsiung Medical University, Kaohsiung 80708, Taiwan; 7 Department of Biomedical Science and Environmental Biology, Kaohsiung Medical University, Kaohsiung 80708, Taiwan

**Keywords:** intracerebral hemorrhage, miRNA-195-5p, MMP, apoptosis

## Abstract

Intracerebral hemorrhage (ICH) remains a devastating disease with high mortality, and there is a lack of effective strategies to improve functional outcomes. The primary injury of ICH is mechanical damage to brain tissue caused by the hematoma. Secondary injury, resulting from inflammation, red cell lysis, and thrombin production, presents a potential target for therapeutic intervention. Inflammation, crucial in secondary brain injury, involves both cellular and molecular components. MicroRNAs (miRNAs) are vital regulators of cell growth, differentiation, and apoptosis. Their deregulation may lead to diseases, and modulating miRNA expression has shown therapeutic potential, especially in cancer. Recent studies have implicated miRNAs in the pathogenesis of stroke, affecting endothelial dysfunction, neurovascular integrity, edema, apoptosis, inflammation, and extracellular matrix remodeling. Preclinical and human studies support the use of miRNA-directed gene modulation as a therapeutic strategy for ICH. Our study focused on the effects of miR-195 in ICH models. Neurological tests, including the corner turn and grip tests, indicated that miR-195 treatment led to improvements in motor function impairments caused by ICH. Furthermore, miR-195-5p significantly reduced brain edema in the ipsilateral hemisphere and restored blood–brain barrier (BBB) integrity, as shown by reduced Evans blue dye extravasation. These results suggest miR-195-5p’s potential in attenuating ICH-induced apoptosis, possibly related to its influence on MMP-9 and MMP-2 expression, enzymes associated with secondary brain injury. The anti-apoptotic effects of miR-195-5p, demonstrated through TUNEL assays, further underscore its therapeutic promise in addressing the secondary brain injury and apoptosis associated with ICH. In conclusion, miR-195-5p demonstrates a significant neuroprotective effect against ICH-induced neural damage, brain edema, and BBB disruption, primarily through the downregulation of MMP-9 and MMP-2. Our findings indicate that miR-195-5p holds therapeutic potential in managing cerebral cell death following ICH.

## 1. Introduction

Intracerebral hemorrhage (ICH) presents a significant threat due to its high fatality rates [1]. Currently, there is a lack of treatments that can reduce mortality or enhance survivors’ quality of life. With the advent of computed tomography (CT), medical professionals identified a hyperperfused region adjacent to the hematoma in the brain. This region has been labeled as luxury perfusion, relative focal hyperperfusion, and focal hyperemia. While metabolic acidosis is posited as a possible reason for this occurrence, the clinical manifestations of hyperperfusion following ICH still lack clarification. The neurological impact post-ICH is twofold: Initially, the expanding hematoma exerts mechanical pressure. After the clot solidifies, inflammation and edema contribute to additional brain tissue harm. Processes linked with clot development seem to instigate inflammation and edema. Wagner et al. observed that lactate levels rose in swollen brain tissue near ICH sites without a corresponding adenosine triphosphate (ATP) drop, pointing towards aerobic glycolysis possibly triggered by the uptake of hematoma-derived glutamate by astrocytes [2].

Matrix metalloproteinases (MMPs), a set of zinc-dependent endopeptidases, can degrade elements of the extracellular or neurovascular matrix, leading to neurovascular trauma [3]. MMP-2 and MMP-9, predominantly found in brain vessels [4], can be stimulated by free radicals via the nuclear factor kappa B (NF-κB) route, resulting in heightened vascular leakage and brain swelling. Specifically, MMP-9 plays a crucial role during the acute phase post-ICH, and inhibiting MMP activity or eliminating the MMP-9 gene during this time has shown potential in treating subsequent brain damage [5]. Poloxamer 188, a surfactant, has been shown to guard against ICH by obstructing blood–brain barrier (BBB) disruptions via the NF-κB-MMP-mediated degradation of tight junction proteins [6], positioning it as a viable candidate for attenuating post-hemorrhage inflammatory responses [7].

MicroRNAs (miRNAs) are short, non-coding RNA strands spanning 20–25 base pairs that oversee gene expression during post-transcription by interacting with the target messenger RNA in the 3′ untranslated region [8,9]. Within the cell nucleus, miRNAs start as pri-miRNA, later morphing into a short pre-miRNA structure [10]. This pre-miRNA then transitions into mature miRNA in the cytoplasm. Mature miRNAs can pair with one or multiple mRNAs, leading to protein synthesis inhibition or mRNA splitting. Given their role in cell growth and differentiation [9,11,12], any miRNA deregulation can be linked to diseases. Much emphasis has been laid on miRNAs in cancer research, given their involvement in tumorigenesis [13,14,15], and manipulating miRNA expression offers therapeutic promise in cancer treatment [11,12,16].

Stroke, a major global killer, can be classified into ischemic and hemorrhagic types, both tied to glial harm and neuroinflammation. Recent investigations indicate multiple miRNAs and associated genes play a part in the development of stroke, affecting endothelial function, neurovascular balance, edema, apoptosis, inflammation, and extracellular matrix reshaping [15,17,18,19,20,21]. For instance, miR-145 has been identified as a vascular smooth muscle cell marker, and miR-29b has been linked to increased cardiovascular risk due to its effect on the DNA methylation of MMP-2/MMP-9 genes [20]. More insights are available in recent review articles [7,22].

In relation to ICH, studies in animal models have highlighted miRNA expression trends [19]. Furthermore, several miRNAs, when expressed differently in the blood plasma of ICH patients, might act as indicators to anticipate hematoma growth post-ICH [23]. Changes in the levels and distribution of multiple inflammation-associated miRNAs in plasma due to ICH have been documented [24]. Additionally, bioinformatics evaluations of the current data indicate a concentration of upregulated miRNAs in inflammatory disease processes and reactions post-ICH. These findings align with prior research on the expression of genes associated with inflammation, demonstrating a multifaceted inflammatory reaction following experimental ICH [25]. Research from both laboratory animal models and human subjects indicating mRNA expression patterns, the suppression of cell life-preserving pathways, and heightened expression of inflammatory genes, provide insights into a potential treatment approach for ICH via miRNA-driven gene adjustment [26,27].

miR-195-5p, a member of the miR-15/107 family, has been extensively studied for its role as a tumor suppressor in various cancers [28]. Beyond oncology, miR-195-5p has significant implications in several other medical conditions. It has been reported to be downregulated in serum samples from patients with essential hypertension and type 2 diabetes mellitus and it shows clinical significance by targeting dopamine receptor D1 [29]. Additionally, miR-195-5p expression is notably downregulated in severe preeclampsia placentas compared to normal pregnant controls [30]. In the context of neurological diseases, miR-195-5p has been shown to regulate the PTEN-AKT signaling pathway, which is crucial for cerebral ischemia–reperfusion injury [29]. Furthermore, miR-195-5p has been found to confer protection against renal ischemia–reperfusion injury by targeting vascular endothelial growth factor A, thereby inhibiting inflammation and oxidative stress and attenuating acute kidney injury [31]. These findings suggest a broader role for miR-195-5p in mitigating various forms of tissue damage through its anti-inflammatory and anti-apoptotic effects. Based on previous studies, miR-195-5p is hypothesized to have neuroprotective abilities against ICH-induced necrotic neuronal cell death, potentially mediated through the MMP-9/MMP-2 pathways. It may be effective in the management of ICH-induced neurotoxicity not only via its neuroprotective effect but also via anti-apoptosis and anti-inflammatory effects after ICH. This study is designed to reach a logical conclusion to the above hypothesis that miR-195-5p serves as an alternative strategy in treating patients with ICH.

## 2. Materials and Methods

### 2.1. Animal Preparation and Surgical Preparation: Induction of Intracerebral Hemorrhage

Sprague–Dawley rats, aged 7 weeks and weighing between 200 and 250 g, were utilized for the study. The animals had unrestricted access to both food and water. The Center for Laboratory Animals of Kaohsiung Medical University and Use Committee granted approval for all procedures (approval No. 104236). To anesthetize the rats, an intraperitoneal injection of pentobarbital (50 mg/kg) was administered. With the assistance of a heating pad (Harvard Apparatus), the rectal temperature was maintained at 36 ± 1 °C. For the subsequent experiment, a total of 128 male Sprague–Dawley rats were utilized. These rats were categorized into four distinct groups: (1) Control (no ICH with sham surgery); (2) ICH only rats; (3) ICH rats plus NC-mimic; and (4) ICH rats plus miR-195-5p. To induce experimental ICH, a stereotaxic intrastriatal delivery of type IV bacterial collagenase (Sigma-Aldrich, St. Louis, MO, USA) was performed, following the methodologies outlined in studies by Jeong and June [32,33]. In summary, following the intraperitoneal injection of pentobarbital (50 mg/kg), the rats were positioned in a stereotaxic apparatus (David Kopf instruments, Tujunga, CA, USA). Burr holes were then surgically created. Subsequently, a 30-gauge Hamilton syringe needle was carefully introduced into the striatum. The ICH was induced by delivering 1 μL of a solution containing 0.23 μL collagen digestion units of type IV collagenase over 5 min. Upon completing the infusion, the craniotomies were securely closed using bone wax and the rats were individually housed for recovery. Post-ICH, a dosage of 5 nmol/mL/kg of miR-195-5p (5′-UAGCAGCACAGAAAUAUUGGC-3′) was intravenously administered via the tail using in vivo-jetPEI^®^ (from Polyplus Transfection Co., Illkirch, France).

### 2.2. Neurological Tests

#### 2.2.1. Corner Turn Test

Rats were guided into a corner with an angle of 30°. To leave the corner, the rat could turn either left or right. Only turns where the rat fully reared against one of the walls were recorded. Depending on the injury severity, rats might exhibit a bias to turn towards their injured side. The percentage of rightward turns was noted. This test was repeated ten times, ensuring a minimum gap of 30 s between each trial.

#### 2.2.2. Forelimb Grip Strength Test

The forelimb grip strength evaluation was implemented to assess neurological function both prior to and on days 1, 2, 5, 7, 14, and 28 post-ICH. In essence, this evaluation quantified the rats’ gripping power (measured in grams) using a grip strength meter (BIO-GS3, BioSeb, Vitrolles, France). During the test, a rat’s forelimbs were swiftly yet gently positioned on the grip bar. Subsequently, the rat was pulled horizontally back until it let go of the bar. A blinded observer, unaware of the rat’s treatment group and identity, gauged the performance. The grip strength, when assessed, was adjusted according to the rat’s body weight and expressed as a percentage relative to the control group.

### 2.3. Measurement of Brain Water Content

Three days post-surgery, six rats from each group were anesthetized and euthanized through decapitation. Their brains were promptly extracted, and each brain was bisected along the central axis. Following this, the cerebellum was detached from every brain. Brain samples were instantly weighed to determine their wet weight using an electronic analytical balance. Subsequently, these samples were dried in a gravity oven set at 100 °C for a period of 24 h to ascertain their dry weights. The brain’s water content was represented as a proportion of its wet weight. The equation employed to determine this water content was: (wet weight − dry weight)/(wet weight).

### 2.4. Evaluation of Blood–Brain Barrier (BBB) Permeability

The BBB permeability was evaluated through the Evans blue dye exudation method, with four rats examined from each group. Forty-eight hours post-ICH, a solution of 2% Evans blue dissolved in normal saline (2 mL/kg body weight) was administered via the tail vein. The dye was allowed to circulate for 30 min. Subsequently, the rats were anesthetized and euthanized by decapitation. After perfusing with 200 mL of saline, the brain was extracted and separated into the right and left hemispheres. Each brain specimen was weighed, then combined with ice-cold phosphate-buffered saline (PBS) in a 1:2 ratio (sample weight to PBS). These samples underwent homogenization followed by centrifugation at 15,000 rpm for 30 min at 4 °C. The resulting supernatant was partitioned into aliquots. For every 500 μL aliquot, an equivalent volume of 50% trichloroacetic acid was added. This mixture was incubated overnight at 4 °C before undergoing another round of centrifugation at 15,000 rpm for 30 min at 4 °C. The Evans blue dye was then extracted and its concentration assessed using a spectrophotometer (Multiskan™ FC Microplate Photometer, Thermo Fisher Scientific, Waltham, MA, USA) set at 610 nm. The quantification was based on a predetermined standard curve.

### 2.5. Evaluation of Hematoma Value (Spectrophotometric Assay)

The volume of hemorrhage was determined using a spectrophotometric assay, with four rats tested per group. Forty-eight hours post-ICH, the rats were anesthetized, then euthanized by decapitation. Following a 200 mL saline perfusion, the brain was extracted and separated into its right and left hemispheres. Each brain specimen was weighed and subsequently combined with ice-cold PBS at a ratio of 1:2 (sample weight to PBS). These samples were then homogenized and centrifuged at 15,000 rpm for 30 min at 4 °C. The resulting supernatant was carefully collected. For assessing the hematoma, 40 μL of the sample supernatant was combined with 160 μL of Drabkin’s reagent (Sigma-Aldrich) and allowed to react for 15 min at room temperature. This interaction converted all the hemoglobin forms into cyanomethemoglobin. The optical density was gauged at 540 nm using a spectrophotometer (Multiskan™ FC Microplate Photometer, Thermo Fisher Scientific). Hemoglobin levels in the supernatant were then estimated based on a reference curve derived from known hemoglobin concentrations. The methodology for measuring hematoma volume using hemoglobin concentration was adapted from the study by Asahi et al. [34].

### 2.6. Tissue Preparation for TUNEL Stain

Three days post-operation, rats from each group (*n* = 6) were re-anesthetized. A heart perfusion was performed using 250 mL of cold saline followed by 250 mL of 4% paraformaldehyde in 0.1 mol/L phosphate-buffered saline. After a 24 h fixation in 4% paraformaldehyde, the brains were placed in 30% sucrose for another 24 h for cryoprotection. Subsequently, the brains were sectioned into 12 μm slices using a cryostat, a process detailed in other studies. For the in situ detection of DNA fragmentation, the brain sections underwent a TUNEL (Roche, Basel, Switzerland) procedure using the in situ cell death detection kit. Quantitative analysis of the cells was carried out with the ImageJ (version: 1.51e) software from the National Institutes of Health.

### 2.7. Western Blotting Analysis

To investigate the role of miR-195-5p in ICH-induced injury, the levels of MMP-9 and MMP-2 were assessed using Western blotting. Twenty-four hours post-operation, the rats were euthanized by decapitation and their brains promptly removed (*n* = 6 for each group). Homogenates of the ipsilateral hemisphere were centrifuged at 13,000 rpm for 20 min. Subsequently, 30 μg of protein extracts were electrophoresed on either 8% or 10% sodium dodecyl sulfate–polyacrylamide gel electrophoresis (SDS-PAGE) and transferred onto polyvinylidene fluoride (PVDF) membranes. These membranes were first blocked with 5% skim milk in TBST (containing 50 mM Tris pH 7.5, 0.15 mM NaCl, and 0.05% Tween 20) and subsequently incubated with anti-MMP-9 (1:500; Merk Millpore, Burlington, VT, USA) and anti-MMP2 (1:500, Merk Millpore) antibodies in TBST at 4 °C overnight. The membranes were then exposed to horseradish peroxidase (HRP)-conjugated secondary antibodies in TBST for 1 h at room temperature. An anti-beta-actin antibody (1:20,000; Sigma) served as the internal control. Visualization of the bands was achieved using enhanced chemiluminescence (PerkinElmer, Waltham, MA, USA). The resulting blots were digitally captured (MiniChemi500, SageCreation, Taipei, Taiwan) and quantified using ImageJ software.

### 2.8. Matrix Metalloproteinase Gelatin Zymography

To validate the reduced activity of MMP-9 and MMP-2, brain tissue samples were examined for MMP-9 and MMP-2 gelatinase activities using zymography. Twenty-four hours post-operation, gel zymography was conducted (*n* = 6 in each group). Protein samples, prepared similarly to those in the Western blot analysis, were employed. Equal portions (30 μg) of these protein extracts were run on a 10% tris-glycine gel infused with 0.1% gelatin as a substrate (Invitrogen, Waltham, MA, USA). Post-electrophoresis, the gel underwent renaturation and was then incubated in a developing buffer at 37 °C for a duration of 24 h. Subsequently, the gel was stained with SimpleBlue Safestain (Invitrogen) for an hour, followed by a destaining process. Clear areas on the gel indicated gelatinolytic activity.

### 2.9. Statistical Analysis

Continuous values are presented as mean ± standard deviation. A two-tailed Student’s *t*-test was utilized to compare values between paired groups. For comparisons among multiple experimental groups, either Student’s *t*-test or one-way analysis of variance (ANOVA, using SPSS version 20.0, IBM SPSS Statistics) was employed. A *p*-value of less than 0.05 was deemed statistically significant.

## 3. Results

### 3.1. Neurological Tests

**Figure 1:**
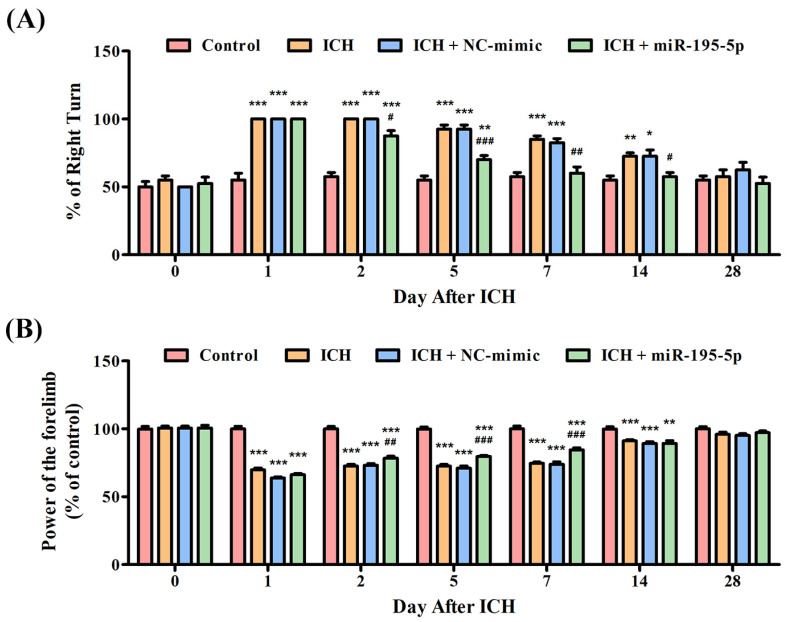
Evaluation of neurobehavioral recovery post-ICH with miR-195-5p treatment. This study showed the percentages of right turn and power of control performed by rats in four groups (Control, ICH, ICH + NC-mimic, and ICH + miR-195-5p treatment) in the (**A**) corner turn test and (**B**) grip test, conducted over 28 days following ICH induction by collagenase infusion. * *p* < 0.05, ** *p* < 0.01, and *** *p* < 0.001 compared with the control group. # *p* < 0.05, ## *p* < 0.01, and ### *p* < 0.001 compared between the ICH + NC-mimic group and the miR-195-5p-treated group.

To confirm the neuroprotective function of miR-195-5p or its ability to alleviate the neural damage induced by ICH, the corner turn test and the grip test were conducted. In the corner turn test, the initial readings on day 0 show uniform performance across all groups, but as time progresses, those subjected to ICH exhibit a marked decrease in the percentage of right turns, indicating a deficit caused by the hemorrhage. In addition, the group treated with miR-195-5p demonstrates a relative improvement, suggesting a therapeutic benefit (Figure 1A). Similarly, in the grip test, all groups initially exhibit comparable grip strengths. However, post-ICH, a significant decline is observed in the ICH group’s grip strength, a decline that persists but shows signs of recovery over the course of the study. The group receiving miR-195-5p treatment shows a significant early improvement in grip strength, especially on days 1 and 2 after ICH induction, compared to the ICH + mimic group without the treatment. This suggests that miR-195-5p may exert a protective or restorative effect shortly after the injury, but this effect appears to diminish over time (Figure 1B). These data suggest that while ICH causes a noticeable impairment in motor function, as evidenced by both the corner turn and grip tests, the application of miR-195-5p has the potential to ameliorate these deficits, particularly in the early stages following the hemorrhage.

### 3.2. Brain Water Content

**Figure 2:**
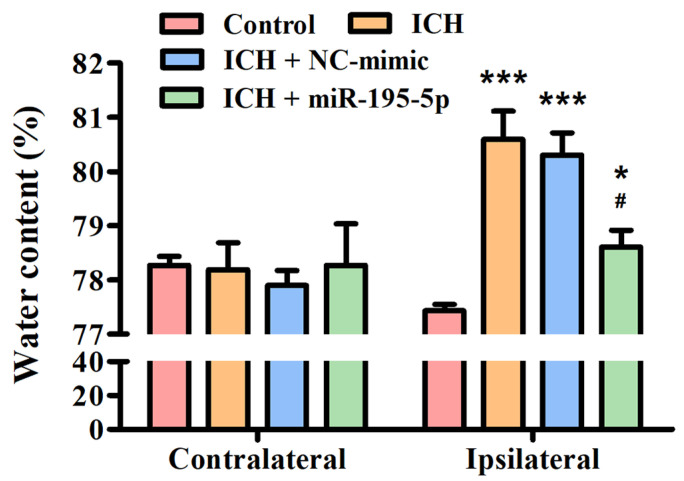
Brain edema assessment in the rat model post-ICH treatment. This study showed the percentage of water content in the contralateral and ipsilateral brain hemispheres among four groups: Control, ICH, ICH + NC-mimic, and ICH + miR-195-5p. The analysis was conducted after inducing ICH by collagenase infusion into the caudate nucleus of rats. * *p* < 0.05 and *** *p* < 0.001 compared with control group. # *p* < 0.05 compared with the ICH + NC-mimic group. (*n* = 6 in each group).

In order to investigate the potential effects of miR-195-5p on brain edema following an ICH, an experiment focusing on water content analysis was conducted in both the contralateral and ipsilateral hemispheres of the brain at 72 h after collagenase infusion. This study aimed to discern whether miR-195-5p could mitigate the increase in water content, a key indicator of brain edema, which is a common and detrimental consequence of ICH. In the contralateral hemisphere, all groups, which include the Control, ICH, ICH + NC-mimic, and ICH + miR-195-5p, show similar water content percentages, indicating no significant edema or difference in edema between the groups on the side of the brain opposite to the ICH at 72 h after infusion of collagenase. In the ipsilateral hemisphere, there is a clear distinction between the groups. The ICH group shows a significantly higher water content compared to the control group, indicating a pronounced edema associated with the hemorrhagic event at 72 h after infusion of collagenase (*p* < 0.01) (Figure 2). This suggests that the brain’s response to the ICH involves a substantial increase in water content on the affected side. However, when treated with miR-195 (ICH + miR-195-5p group), the water content in the ipsilateral hemisphere is significantly reduced compared to the untreated ICH + mimic group. This reduction is statistically significant, suggesting that miR-195-5p treatment is effective in reducing the brain edema that follows ICH. The water content in the miR-195-5p-treated group is also significantly different from the control group, but the difference is less pronounced (*p* < 0.05) (Figure 2). This indicates that while the treatment does not bring the water content back to normal levels, it has a substantial therapeutic effect. The ICH + NC-mimic group does not show a significant difference from the control group, which implies that the NC-mimic treatment does not significantly impact edema when compared to the normal condition.

### 3.3. miR-195-5p Improves the Blood–Brain Barrier Permeability after ICH

**Figure 3:**
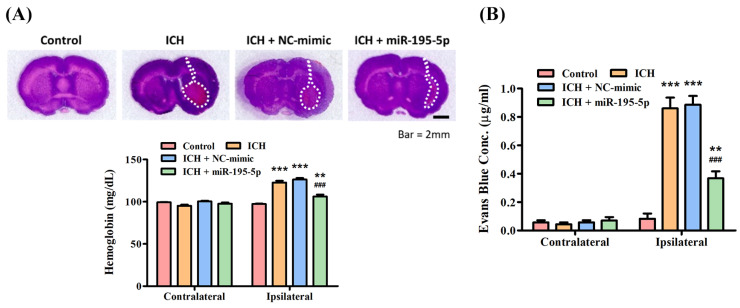
Assessment of hematoma volume and blood–brain barrier integrity post-ICH. The effects of ICH and the therapeutic intervention with miR-195-5p on brain injury. (**A**) Hemoglobin content measurements, indicative of hematoma volume, reveal that while the ICH and ICH + NC-mimic groups show an increase in the ipsilateral hemisphere suggestive of larger hematomas, miR-195-5p treatment significantly reduces this volume, indicating its potential to alleviate hemorrhage-associated brain damage. (**B**) The assessment of BBB integrity through Evans blue dye extravasation demonstrates heightened permeability in the ICH groups, which is notably decreased following treatment with miR-195-5p. (** *p* < 0.01 and *** *p* < 0. 001 different from control, ### *p* < 0. 001 compared with ICH + NC-mimic) (*n* = 4 in each group).

In order to assess BBB permeability, the Evans blue dye extravasation assay was performed on day 3 after ICH. The hematoma value assay displays the hemoglobin levels in both the contralateral and ipsilateral hemispheres of the brain across different groups: Control (97.2581 ± 1.3242 mg/dL), ICH (122.6613 ± 3.9791 mg/dL), ICH + NC-mimic (126.7908 ± 3.4164 mg/dL), and ICH + miR-195-5p (106.6290 ± 4.4898 mg/dL). Hemoglobin levels in the contralateral hemisphere remain relatively consistent across all groups, indicating no significant bleeding or hematoma formation on the side of the brain opposite to the induced hemorrhage (Figure 3A). However, in the ipsilateral hemisphere, where the ICH was induced, there is a notable increase in hemoglobin levels for the ICH and ICH + NC-mimic groups, signifying the presence of a hematoma (Figure 3A). The hemoglobin concentration is notably less in the ICH + miR-195-5p group compared to the ICH + mimic group, marked by statistical significance (* *p* < 0.05) (Figure 3A), indicating that miR-195-5p treatment effectively reduces the size of the hematoma. The Evans blue assay evaluates the integrity of the BBB by measuring the extravasation of the Evans blue dye, which can only occur when the BBB is compromised. Three days after ICH, the assay shows a significant increase in dye extravasation in the ipsilateral hemisphere of the ICH (0.8601 ± 0.1525 μg/mL) and ICH + NC-mimic (0.8860 ± 0.1082 μg/mL) groups compared to the control (0.0824 ± 0.0701 μg/mL) (** *p* < 0.01) (Figure 3B). This confirms that ICH leads to BBB disruption. In contrast, the ICH + miR-195-5p (0.3674 ± 0.0923 μg/mL) group exhibits a substantial decrease in dye extravasation compared to the ICH + mimic group (# *p* < 0.05) (Figure 3B), suggesting that miR-195-5p treatment significantly restores BBB integrity. These data suggest that ICH causes significant hematoma formation and BBB disruption, effects that can be mitigated by the administration of miR-195-5p. This compound shows promise in reducing the physical size of the hematoma and in protecting the integrity of the BBB, which are crucial for reducing secondary brain injury after ICH.

### 3.4. miR-195-5p Inhibits Neuron Apoptosis after ICH

**Figure 4:**
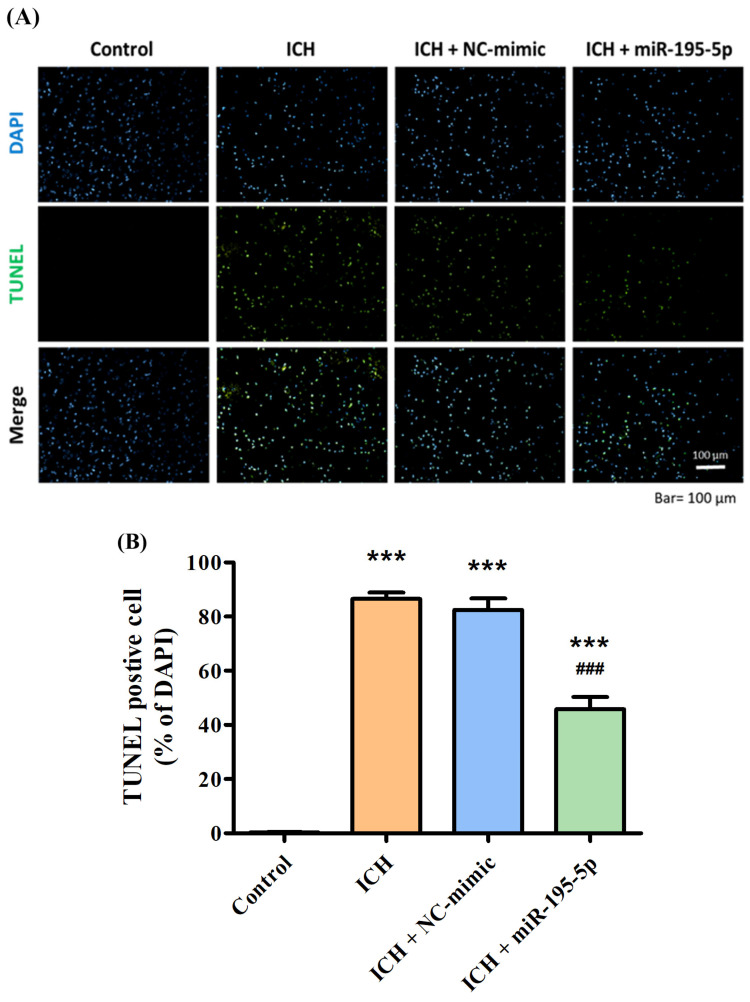
TUNEL assay demonstrating apoptotic cell death in response to ICH and treatment with miR-195-5p. The apoptotic response in brain tissue following ICH and the effect of miR-195-5p treatment, as visualized by fluorescence microscopy and quantified through TUNEL staining. (**A**) In the images, DAPI staining marks all cell nuclei in blue, while TUNEL staining identifies apoptotic cells in green, with merged images providing a comprehensive view of apoptosis within the total cell context. (**B**) The control group maintains minimal apoptosis, contrasting with the ICH and ICH + NC-mimic groups, which show increased numbers of TUNEL-positive cells. The ICH + miR-195-5p group reveals a marked reduction in apoptotic cells, underscoring the protective role of miR-195-5p in mitigating cell death associated with ICH. (*** *p* < 0.001 compared with control, ### *p* < 0.001 compared with ICH + NC-mimic) (*n* = 4 in each group).

To demonstrate the neuroprotective ability of miR-195-5p, the TUNEL assay was used in this study. The control group shows a healthy cellular environment with a clear DAPI signal and an absence of TUNEL staining, indicating no significant apoptosis (TUNEL-positive cells: 0.33) (Figure 4A). In contrast, the ICH group shows a considerable increase in TUNEL-positive cells, highlighting the extent of apoptosis that occurs in response to the hemorrhagic injury 72 h after collagenase infusion (TUNEL-positive cells: 86.58) (Figure 4A). The ICH + NC-mimic group presents a similar level of TUNEL positivity, suggesting that the NC-mimic (TUNEL-positive cells: 82.44) does not confer significant neuroprotection against the apoptosis induced by ICH (Figure 4B). Significantly, the ICH + miR-195-5p group (TUNEL-positive cells: 45.32) shows a visibly reduced level of TUNEL staining compared to the ICH + mimic group (Figure 4B). This indicates that miR-195-5p provides a neuroprotective effect, reducing the number of cells undergoing apoptosis in the aftermath of the hemorrhage.

### 3.5. miR-195-5p Inhibits MMP-9/MMP-2 Enzymatic Activity after ICH

**Figure 5:**
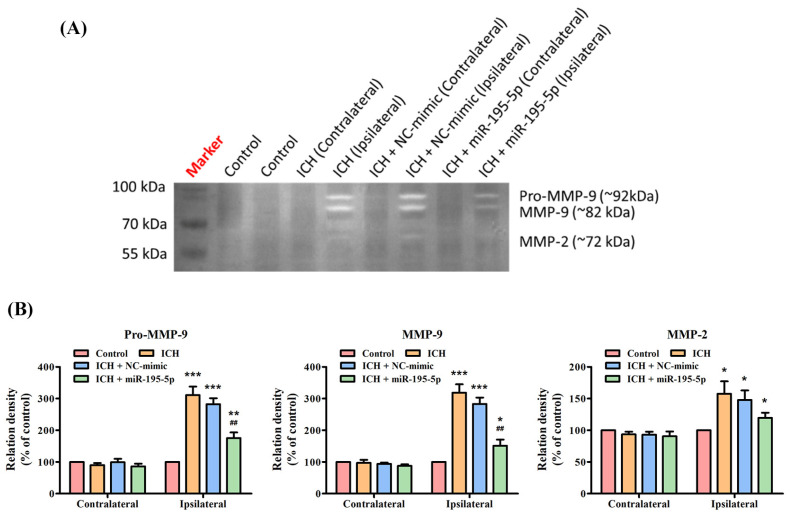
Matrix metalloproteinase zymography assessing MMP activity post-ICH and miR-195-5p treatment. The impact of ICH on the activity of various MMPs and the modulatory effects of miR-195-5p treatment. (**A**) The zymogram reveals the activity levels of Pro-MMP-9, MMP-9, and MMP-2, with samples from both unaffected (contralateral) and affected (ipsilateral) brain hemispheres across the control, ICH, and ICH-treated groups with either NC-mimic or miR-195-5p. It highlights a significant upregulation of Pro-MMP-9 and MMP-9 activities in the ipsilateral hemisphere following ICH, which is notably diminished upon treatment with miR-195-5p, indicating its therapeutic potential in mitigating MMP-related pathologies post-ICH. (**B**) The bar graphs present a quantitative comparison of these enzymes’ activities, showing relative enzyme densities to the control and demonstrating the specific reduction in MMP activity (particularly for Pro-MMP-9 and MMP-9) associated with miR-195-5p treatment. (*** *p* < 0.001, ** *p* < 0.01, and * *p* < 0.05 compared with the control group, ## *p* < 0.01 compared with the ICH + NC-mimic group) (*n* = 4 in each group).

For the Pro-MMP-9 and MMP-9 activation, the ICH group shows a significant increase in these enzymes’ activity in the ipsilateral hemisphere, indicating a response to the hemorrhagic injury (Figure 5A). However, when miR-195-5p is administered, there is a notable reduction in the levels of these enzymes, particularly for MMP-9, which is significantly lower than the levels observed in the untreated ICH group. This reduction is highlighted by the statistical significance in the comparisons with the control group (*p* < 0.01) and the ICH group (*p* < 0.01) (Figure 5B). However, for MMP-2, the variation across all groups, including the treatment group with miR-195-5p, is less pronounced and there are no statistically significant changes indicated in the ipsilateral hemisphere among the different groups (Figure 5B). These findings suggest that miR-195-5p administration has a significant impact on reducing MMP-9 activity, which is elevated after ICH. This could indicate that miR-195-5p plays a role in modulating the inflammatory response and tissue remodeling that occurs after a hemorrhagic brain injury, potentially contributing to a protective effect against further damage to the brain tissue.

### 3.6. miR-195-5p Reduces the Protein Expression of ICH-Induced MMP-9 and MMP-2

**Figure 6:**
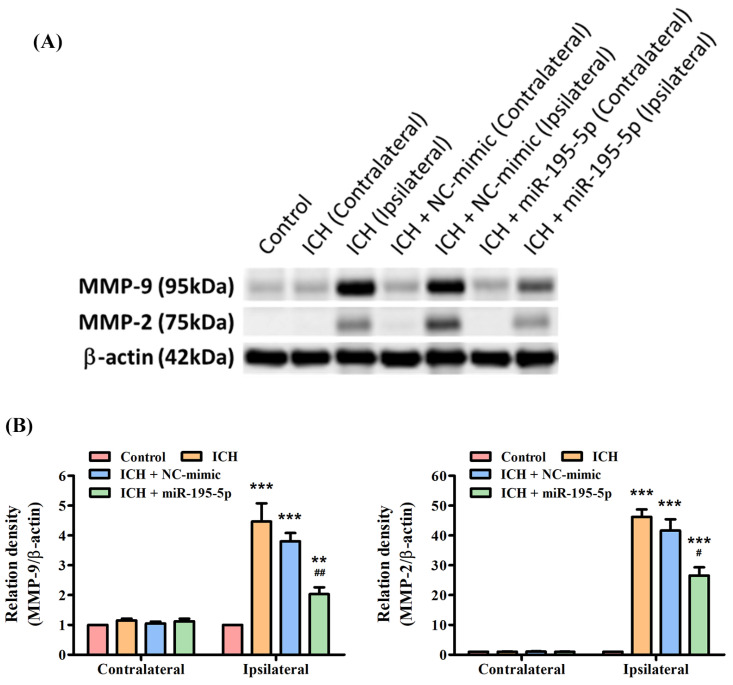
Differential expression of MMP-9 and MMP-2 post-ICH and miR-195-5p intervention as revealed by Western blotting. The effects of ICH and miR-195-5p treatment on MMP-9 and MMP-2 protein expression. (**A**) Western blots reveal the presence of MMP-9 and MMP-2 proteins, alongside β-actin as a loading control, with samples taken from the control group, the ICH group, and ICH groups treated with either NC-mimic or miR-195-5p in both the unaffected contralateral and the affected ipsilateral brain hemispheres. (**B**) The quantitative assessment shows a significant increase in MMP-9 expression in the ipsilateral hemisphere following ICH, which is notably decreased after miR-195-5p treatment, suggesting the treatment’s effectiveness in reducing the upregulated levels of MMP-9 associated with ICH. MMP-2 expression also rises post-ICH but is somewhat decreased by miR-195-5p, with levels still above those of the control, reflecting a differential response to the treatment. (*** *p* < 0.001 and ** *p* < 0.01 compared with the control group, # *p* < 0.05 and ## *p* < 0.01 compared with the ICH + NC-mimic group) (*n* = 4 in each group).

The Western blot indicated that both MMP-9 and MMP-2 proteins are significantly upregulated in the ipsilateral hemisphere following ICH, as demonstrated by the elevated levels in the ICH and ICH + NC-mimic groups compared to the control group (Figure 6A). However, the significant finding is the reduction in the levels of both MMP-9 and MMP-2 in the ipsilateral hemisphere of the ICH group treated with miR-195-5p (Figure 6B). This reduction is also statistically significant compared to the ICH + mimic group, indicating that miR-195-5p has a downregulatory effect on the expression of these MMPs (Figure 6B). The results suggest that while ICH induces an upregulation of MMP-9 and MMP-2, which are implicated in the degradation of the extracellular matrix and could contribute to secondary injury after hemorrhage, treatment with miR-195-5p can effectively mitigate this upregulation. Thus, miR-195-5p may have a protective role in the aftermath of ICH, potentially by modulating the activity of MMPs that contribute to the pathophysiology of brain injury.

## 4. Discussion

Our study demonstrated that miR-195-5p treatment led to significant improvements in motor function, as evidenced by the corner turn test and grip test results. miR-195-5p significantly reduced brain edema in the ipsilateral hemisphere and restored blood–brain barrier (BBB) integrity, as indicated by the reduction in Evans blue dye extravasation. Additionally, miR-195-5p reduced neuronal apoptosis, as shown by the TUNEL assay results, and decreased the activity and expression of MMP-9 and MMP-2, enzymes associated with secondary brain injury post-ICH. These findings suggest that miR-195-5p has a neuroprotective effect against ICH-induced neural damage, primarily through the downregulation of MMP-9 and MMP-2.

Our study utilized different time points to measure various endpoints, which were based on the specific biological processes being assessed. Brain water content and TUNEL staining were measured three days post-ICH, as edema and apoptosis typically peak around this time, providing a clear view of the extent of brain damage and the protective effects of miR-195-5p. BBB permeability and hematoma volume were assessed within 48 h post-ICH to capture the early disruption of the blood–brain barrier and the initial extent of bleeding, which are important for understanding the immediate impact of miR-195-5p treatment on vascular integrity. Western blotting and matrix metalloproteinase gelatin zymography were conducted within 24 h post-ICH to measure the early changes in protein expression and enzyme activity, as early time points are the key points for detecting the initial molecular responses to ICH and the immediate effects of miR-195-5p treatment.

Patients with intracerebral hemorrhage may exhibit impairments in motor planning and execution, leading to an increased number of right turns in the corner turn test and decreased grip strength and coordination in the grip test [35]. The impairments observed in these tests can be attributed to the damage caused by the hemorrhage in the sensorimotor cortex and basal ganglia, which are crucial for motor control and coordination [36,37]. In our study, ICH was induced via stereotaxic intrastriatal administration of type IV bacterial collagenase and had a significant effect on the results of the corner turn test and grip test. However, intravenous injection with miR-195-5p demonstrated a therapeutic benefit and may ameliorate the impairment in motor function.

Hemorrhagic brain damage is a serious condition characterized by bleeding within the brain, often resulting from conditions such as intracerebral hemorrhage [38]. Intracerebral-hemorrhage-induced brain damage can be attributed to the increased expression of matrix metalloproteinases, particularly MMP-9 and MMP-2 [39]. These matrix metalloproteinases play a crucial role in the disruption of the blood–brain barrier, leading to vascular edema and blood extravasation [40]. Both ischemic and hemorrhagic strokes have been associated with altered expression, secretion, and activation of matrix metalloproteinases, which contribute to the disruption of the blood–brain barrier [40]. This disruption subsequently results in vascular edema and blood extravasation, further exacerbating the damage caused by intracerebral hemorrhage [41,42,43]. After an ischemic stroke, the increased expression of matrix metalloproteinases contributes to the disruption of the blood–brain barrier, promoting brain edema and potentially leading to hemorrhage [40].

Among the various subtypes of matrix metalloproteinases, MMP-2 and MMP-9 have been particularly investigated in ischemic stroke [44]. These MMPs have been identified as the principal culprits in the breakdown of the blood–brain barrier and subsequent damage to neurons [41,42,43]. Furthermore, studies have shown that MMP inhibitors can effectively block the breakdown of tight junction proteins and mitigate the effects of ischemic stroke [44].

In the context of intracerebral hemorrhage, evidence from animal models and human studies supports the role of MMP-9 in the disruption of the blood–brain barrier [45]. Direct intracerebral injection of MMP-2 has been shown to cause the opening of the blood–brain barrier and induce intracerebral hemorrhage by disrupting the extracellular matrix [46]. Studies have also demonstrated that in response to conditions such as cerebral-amyloid-angiopathy-related hemorrhage, there is an increase in the expression and activation of MMP-2 and MMP-9 by cerebral vascular smooth muscle cells and endothelial cells. This increased expression and activation of MMP-2 and MMP-9 contribute to the disruption of the blood–brain barrier and ultimately lead to brain damage associated with intracerebral hemorrhage [47]. The increased expression of MMP-9 and MMP-2 induced by intracerebral hemorrhage plays a critical role in the disruption of the blood–brain barrier [40]. This disruption leads to the development of vascular edema and the extravasation of blood, further exacerbating the damage caused by intracerebral hemorrhage [42].

Overall, the increased expression of MMP-9 and MMP-2 induced by intracerebral hemorrhage has a significant impact on the disruption of the blood–brain barrier and the subsequent brain damage [48]. In our study, miR-195-5p improves early motor function recovery and exerts a protective/restorative effect that ameliorates the impairment. Additionally, miR-195-5p has been demonstrated to protect the blood–brain barrier from the increased permeability caused by ICH, decreases the rate of cell death, and lowers the activity of certain enzymes that contribute to inflammation and tissue damage. Overall, miR-195-5p shows the potential as a therapeutic agent that could improve outcomes after a brain hemorrhage by addressing multiple aspects of the injury.

Our study presents several limitations that should be acknowledged. Firstly, the use of a collagenase-induced ICH model, while effective for experimental consistency, may not fully replicate the pathophysiology of spontaneous ICH in humans. This limitation suggests caution when extrapolating our findings to clinical settings. Additionally, the study primarily focused on the early stages of ICH recovery (up to 28 days post-injury), and long-term outcomes of miR-195-5p treatment were not assessed. This restricts our understanding of the sustained efficacy and potential long-term side effects of miR-195-5p treatment. Moreover, while our study highlighted the role of miR-195-5p in regulating MMP-9 and MMP-2 activities, we did not explore other potential molecular pathways and targets that miR-195-5p may influence. This presents a gap in comprehensively understanding the multifaceted mechanisms through which miR-195-5p exerts its neuroprotective effects. In the future, in addition to addressing the aforementioned limitations, research should consider comparative studies using different animal models that better mimic human ICH, as this could enhance the translational relevance of the findings. Additionally, investigating the combined effects of miR-195-5p with other therapeutic agents could provide insights into potential synergistic treatments for ICH. These approaches could help in optimizing therapeutic strategies and improving outcomes for patients with intracerebral hemorrhage.

## 5. Conclusions

The mechanisms of miR-195-5p in attenuating ICH-induced injury may be related to the downregulation of MMP-9 and MMP-2 expression after ICH. The mechanism of miR-195-5p in preventing ICH-induced cell damage needs to be further elucidated. miR-195-5p treatment holds therapeutic promise in the management of cerebral cell death following ICH and is therefore worth further investigation.

## Data Availability

Data are contained within the article.
